#  Eye movements during the reading of narrative and poetic texts. Symposium 6 at the 20th European Conference on Eye Movement Research (ECEM) in Alicante, 21.8.2019. 

**DOI:** 10.16910/jemr.12.7.9

**Published:** 2019-11-25

**Authors:** Arthur Jacobs, Jana Lüdtke, Johanna Kaakinen, Lynn S. Eekhof

**Affiliations:** Center for Cognitive Neuroscience, Department of Experimental and Neurocognitive Psychology, Freie Universität Berlin, Germany; Center for Cognitive Neuroscience, Department of Experimental and Neurocognitive Psychology, Freie Universität Berlin, Germany; University of Turku, Finland; Centre for Language Studies, Radboud University Nijmegen, The Netherlands

**Keywords:** neuropoetics, narrative, Quantitative Narrative Analysis

## Abstract

Despite a wealth of studies using eye tracking to investigate mental processes during vision or reading, the investigation of oculomotor activity during natural reading of longer texts –be it newspaper articles, narratives or poetry– is still an exception in this field (as evidenced by the program of ECEM 2017 in Wuppertal). Following up on our symposium at ECEM 2017, here we bring together eye movement research on natural text reading to report recent progress in a coordinated way sharing data, experiences and software skills in this highly complex subfield. More specifically, in this symposium we will address several challenges faced by an eye tracking perspective on the reading of longer texts which involve a surplus of intervening variables and novel methods to analyze the data. In particular, the following issues will be addressed:

- Which text-analytical and statistical methods are best to deal with the myriad of surface and affective semantic features potentially influencing eye movements during reading of ‘natural’ texts?

- What are the pros and cons of using machine learning assisted predictive modeling as an alternative to the standard GLM/LMM frameworks?

- Which kind of theoretical models can deal with the level of complexity offered by reading longer natural texts?

**Video stream:** https://vimeo.com/358415199

